# Selective Inhibition of Bromodomain-Containing Protein 4 Reduces Myofibroblast Transdifferentiation and Pulmonary Fibrosis

**DOI:** 10.3389/fmmed.2022.842558

**Published:** 2022-03-15

**Authors:** Ksenija Bernau, Melissa Skibba, Jonathan P. Leet, Sierra Furey, Carson Gehl, Yi Li, Jia Zhou, Nathan Sandbo, Allan R. Brasier

**Affiliations:** 1 Department of Medicine, School of Medicine and Public Health (SMPH), University of Wisconsin-Madison, Madison, WI, United States; 2 Department of Pharmacology and Toxicology, University of Texas Medical Branch, Galveston, TX, United States; 3 Institute for Clinical and Translational Research (ICTR), University of Wisconsin-Madison, Madison, WI, United States

**Keywords:** idiopathic pulmonarry fibrosis, myofibroblast, epigenetics, bromodomain and extraterminal domain (BET), bromodomain-containing protein 4

## Abstract

Idiopathic pulmonary fibrosis is a lethal disease driven by myofibroblast expansion. Currently no therapies exist that target the epigenetic mechanisms controlling myofibroblast transdifferentiation, which is responsible for unregulated extracellular matrix (ECM) production. We have recently shown that bromodomain-containing protein 4 (BRD4), an epigenetic regulator that forms a scaffold for nuclear activators and transcription factors, is essential for TGFβ-induced myofibroblast transdifferentiation. However, its role in the development and progression of pulmonary fibrosis *in vivo* has not been established. Here, we evaluate the hypothesis that BRD4 bromodomain interactions mediate myofibroblast expansion and fibrosing disease *in vivo*. C57BL/6J mice challenged with intratracheal bleomycin were systemically treated with a selective allosteric inhibitor of the BRD4 bromodomain 1 (BD1), ZL0591 (10 mg/kg), during the established fibrotic phase (14 days post-bleomycin) in a rigorous therapeutic paradigm. Eleven days after initiation of ZL0591 treatment (25 days post-bleomycin), we detected a significant improvement in blood O_2_ saturation compared to bleomycin/vehicle control. Twenty-eight days post-bleomycin, we observed a reduction in the volumetric Hounsfield Unit (HU) density by micro computed tomography (μCT) in the ZL0591-treated group, as well as a reduction in collagen deposition (hydroxyproline content) and severity of injury (Ashcroft scoring). Myofibroblast transdifferentiation was measured by smooth muscle α-actin (αSMA) staining, indicating a loss of this cell population in the ZL0591-treated group, and corresponded to reduced transcript levels of myofibroblast-associated extracellular matrix genes, tenascin-C and collagen 1α1. We conclude that BRD4 BD1 interactions are critical for myofibroblast transdifferentiation and fibrotic progression in a mouse model of pulmonary fibrosis.

## INTRODUCTION

Idiopathic pulmonary fibrosis (IPF) is a devastating lung disease that leads to death within 3–5 years of diagnosis (Ley et al., 2011). Currently, it is thought that IPF is the result of environmental microinjuries in a host that may be genetically predisposed to aberrant epithelial injury-repair responses. Although complex interactions between epithelial injury-repair signaling, adaptive and innate immunity may initiate and/or propagate the disease, expansion of populations of specialized, activated fibroblasts, myofibroblasts, are primarily responsible for the hallmark fibroblastic foci seen in this disease (Phan, 1996; Tian et al., 2016). Consequently, advancing the understanding of the processes of myofibroblast transdifferentiation and their modification will lead to significant improvements in the outcome of this lethal disease.

Transdifferentiation of mesenchymal fibroblasts in IPF involves the coordinated activation and suppression of hundreds of genes. These processes lead to increased cell motility/invasiveness, contractility and increased expression of ECM proteins (Kis et al., 2011; Torr et al., 2015). A number of lines of work indicate that gene expression programs underlying myofibroblast transdifferentiation involve changes in DNA conformation through covalent modifications of chromatin organization (aka, epigenetic regulation). Of these, acetylation of NH2-terminal lysine residues on core histones has emerged as playing an essential role in driving myofibroblast transdifferentiation (Tzouvelekis and Kaminski, 2015; Duong and Hagood, 2018). Mechanistically, histone acetylation disrupts the ionic interactions between DNA and the histone core, enabling transcriptional machinery’s access to the DNA. Acetylated histones also serve as anchors for binding nuclear bromodomain (BD)-containing proteins, including p300/CBP and members of the bromodomain and extraterminal domain (BET) family. BD-containing protein binding further enhances histone acetylation and serves as a scaffold to recruit transcriptional machinery to inducible genes (Nakamura et al., 2007; Huang et al., 2009; Devaiah et al., 2012). While p300/CBP has been implicated in TGFβ-induced collagen (Col) expression, a non-selective inhibitor of all members of the BET family, JQ1-when given at pharmacological concentrations during the acute inflammatory phase-diminished bleomycin-induced pulmonary fibrosis in mice (Tang et al., 2013). This suggests an important role for BET proteins in the development of pulmonary fibrosis, however, the individual role of BET family members in myofibroblast transdifferentation and IPF remains unclear.

We previously discovered that bromodomain-containing protein 4 (BRD4), one of the most highly expressed BET family members in lung cells, complexes with Smad3 during TGFβ-induced NADPH oxidase 4 (Nox4) transcription to elicit dermal fibroblast to myofibroblast transdifferentiation *in vitro* (Ijaz et al., 2017). Not only does BRD4 mediate transcriptional elongation (Tian et al., 2019a), but it also participates in unique histone acetylation reactions, particularly acetylation of histone H3 Lys (K) 122 (Devaiah et al., 2016a). H3K122 acetylation is a modification that disrupts nucleosomal positioning, further facilitating transcriptional elongation. *In vivo*, we also found that BRD4 is responsible for epithelial to mesenchymal transition in a repetitive polyinosinic:polycytidylic acid ([poly (I:C)]–induced model of airway remodeling (Ijaz et al., 2017; Tian et al., 2019a). However, the role of BRD4 in progression of IPF has not been elucidated. To address this unresolved question, we developed a third generation, highly selective, allosteric inhibitor against BRD4, ZL0591, that has morpholine and 4-methylpiperazine moieties in its backbone, which improve its half-life to 11.8 h after IV administration, facilitating *in vivo* use (Brasier and Zhou, 2020; Liu et al., 2020; Zhou et al., 2021). Importantly, ZL0591 does not target the highly conserved BD’s central binding pocket and is therefore capable of specifically binding to BRD4 BD1 with ~100 nM affinity, while demonstrating 10-fold lower affinity for BRD4 BD2 and BRD3 (Liu et al., 2020; Zhou et al., 2021). In addition, ZL0591 administration disrupts poly (I:C)-induced H3K122 acetylation, confirming that ZL0591 directly engages with its BRD4 target *in vivo* (Liu et al., 2022).

In this study, we applied this selective inhibitor to test whether BRD4 BD1 mediated pulmonary fibrosis in the bleomycin mouse model of the disease. We found that ZL0591, administered in a *bone fide* treatment paradigm during the peak of fibrosis (Moeller et al., 2008; Jenkins et al., 2017), significantly decreases lung fibrosis, collagen content, and myofibroblast transdifferentiation. This work reveals that BRD4 BD1 plays a critical role in the progression of bleomycin-induced pulmonary fibrosis in mice and that targeting this epigenetic regulator may be a useful strategy for anti-fibrotic therapies in the human disease.

## MATERIALS AND METHODS

### Materials

The BRD4 selective BD1 competitive inhibitor, ZL0591, was synthesized as previously described and determined to be >99% pure by HPLC (Liu et al., 2018; Zhou et al., 2021). The compound was initially dissolved in DMSO and diluted into 10% hydroxypropyl β-cyclodextrin (Sigma-Aldrich, St. Louis, MO) in PBS for intraperitoneal (IP) administration.

### Mice

Mouse protocols were reviewed and approved by the University of Wisconsin-Madison School of Medicine and Public Health Institutional Animal Care and Use Committee (IACUC) and adhered to guidelines set by National Institutes of Health (NIH) and Association for Assessment and Accreditation of Laboratory Animal Care (AAALAC).

Eleven-week-old C57BL/6J female mice (Jackson Laboratories, Bar Harbor, ME) were housed in groups of five or less, in static cages with Diamond Dri paper pellet bedding (Inovive, San Diego, CA) and unlimited access to laboratory chow and water. The specific pathogen-free level 3, animal biosafety level 1 facility was under controlled temperature (~74°F), humidity (~30%) and illumination (12 h light/12 h dark cycle). Animals received daily veterinary health checks and weekly cage changes.

For bleomycin instillation, mice were anesthetized with IP administration of ketamine (100 mg/kg, Zoetis Inc., Parsippany-Troy Hills, NJ) and xylazine (15 mg/kg, Akorn Pharmaceuticals, Lake Forest, IL) prior to delivery of a single intratracheal dose of bleomycin (1 U/kg, Teva Pharmaceutical Industries, Israel) dissolved in 50 μL 0.9% normal saline (NS, B. Braun Medical Inc., Bethlehem, PA). Control animals were treated with an intratracheal dose of 50 μL of NS and were kept in separate cages. Mouse weight and survival after treatment were documented daily until endpoint on days 14, 21 or 28 post-bleomycin. Cages carrying five mice/cage were allocated to each group [one cage for NS-treated group and two cages for each of the bleomycin-treated groups to account for previously observed mortality rates post-bleomycin in C57Bl/6J females (Bernau et al., 2015; Bernau et al., 2021)]. Daily IP injections of ZL0591 (10 mg/kg body weight), or vehicle control, were administered to surviving mice from day 14 until 21 and every other day thereafter until endpoint at day 28. At the experimental endpoint (day 14, 21 or 28 post-bleomycin), mice were anesthetized by IP injection of ketamine (200 mg/kg) and xylazine (30 mg/kg) and exsanguinated prior to collection of biological samples.

### Murine Pulse Oximetry

Three days before each endpoint (day 11, 18 or 25 post-bleomycin), murine pulse oximetry was assessed using the MOUSEOX® PLUS (Starr Life Sciences Corp., Oakmont, PA). A small-adult mouse collar-based sensor was placed on awake mice according to the manufacturer’s instruction. In preparation for this, the mice were acclimated to the sensor daily as the animal was weighed.

### *In Vivo* Imaging of Murine Lung Using Micro-Computed Tomography

Mice were initially anesthetized with 4% isoflurane gas and maintained with 2% isoflurane during whole-body micro-computed tomography (μCT) on a Siemens Inveon (Siemens Healthineers, Knoxville, TN) at the University of Wisconsin-Madison Small Animal Imaging and Radiotherapy Facility (SAIRF). Imaging was performed with the following parameters: 360 rotation degrees and rotation steps, binning 4, 250 ms exposure time, 80 kVp, 1 mA and ~105 μm resolution. Reconstructions were performed with no down-sampling using a Shepp-Logan filter and Hounsfield Unit (HU) calibration.

Image analysis assessing μCT lung tissue density was performed using Image Research Workplace similarly as before (Tian et al., 2019a), by selecting and outlining 16 axial lung slices, starting with one immediately superior to the liver in *n* = 4–10 mice/condition. HU values associated with each voxel in the outlined volume were binned into indicated groups with lowest density voxels being in the < −600 HU group and highest density being between 0 and 100 HU. Percent voxels in each group of HU values were calculated based on the total number of voxels in the mouse lung. Individual axial slices were reconstructed in MATLAB and HU represented with a heat map for ease of visualization (Higham and Higham, 2016).

### Hydroxyproline Assay

Assessment of total lung collagen was performed by utilizing a colorimetric hydroxyproline assay, as we have previously done (Bernau et al., 2021). Flash frozen superior, inferior and post-caval lobes of the right lung were homogenized in double distilled H_2_O (ddH_2_O, Sigma Aldrich, St. Louis, MO) using a dounce homogenizer. Lysates were then hydrolyzed in 6 M HCl for 3 h at 120°C. The samples were cooled to room temperature (RT), centrifuged to remove debris, aliquoted in triplicate into 96-well optical plate (Greiner bio-one, 655101) and dried for 45 min at 65°C. Subsequently, samples were incubated in chloramine T solution for 20 min at RT, followed by a 15 min incubation in Ehrlich’s solution at 65°C. Samples were then cooled to RT for 20 min. Absorbance was measured using a colorimetric plate reader at 550 nm. Hydroxyproline content was calculated by utilizing trans-4-Hydroxy-L-proline-generated standard curve (Sigma Aldrich).

### Immunohistochemistry

Mouse left lungs were inflated with 4% neutral buffered formalin (Fisherbrand, Pittsburg, PA) and fixed for 24–48 h before being submerged in 70% ethanol, paraffin embedded and sectioned. Serial lung sections were then subjected to either Masson’s trichrome staining or immunostaining against smooth muscle α-actin (αSMA; rabbit polyclonal antibody from Abcam (ab694), Cambridge, United Kingdom). Negative control stains using the corresponding IgG isotype along with matching secondary antibodies were utilized. Stained slides were scanned using Aperio Digital Pathology Slide Scanner System. Digital snapshots were taken in Aperio ImageScope (Leica Biosystems, Wetzlar, Germany). To quantify αSMA immunostaining, ~ 11 snapshots were taken of the lung parenchyma at ×40 magnification per mouse in *n* = 5–6 mice/condition. The ImageJ immunohistochemistry Toolbox was used to isolate the brown stain, after which images were inverted and their integrated density measured (Schneider et al., 2012; Bernau et al., 2015). High-level magnification (×40) permitted analysis of parenchymal regions that excluded blood vessels and airways so that αSMA + signal from these regions did not bias the results.

### Semiquantitative Scoring of Histologic Fibrosis

Modified Ashcroft scoring to quantify lung fibrosis severity (Hübner et al., 2008) was performed by blinded observers utilizing lung sections that had been subjected to Masson’s trichrome stains, as we have previously done (Hübner et al., 2008). Observers visualized and scored ~10 field of view (FOV) at ×20 magnification per mouse in *n* = 4–10 mice/condition.

### Reverse Transcription-Quantitative PCR (RT-qPCR)

Flash frozen middle lobes of the right lungs were homogenized using a bullet blender with RNA STAT-60 and 0.9–2.0 mm stainless steel RNase-free bullet blender beads before being subjected to the previously described RNA isolation protocol (Bernau et al., 2015; Bernau et al., 2021). RNA was reverse transcribed using SuperScript IV (ThermoFisher Scientific), according to manufacturer’s instructions. cDNA was amplified using TaqMan™ Universal PCR Master Mix (ThermoFisher Scientific) and gene-specific TaqMan™ primers and probes against Collagen 1α1 (*Col1α1*), Vimentin (*Vim*), as well as the housekeeping gene, Peptidylprolyl isomerase A (*Ppia*). The reaction mixtures were subjected to 1 cycle of 2 m at 50°C; 1 cycle of 10 m at 95°C; 45 cycles of 15 s at 95°C, 1 m at 60°C using an AriaMx Realtime PCR System (Agilent Technologies, Santa Clara, CA). For assessment of Tenascin (*Tnc*) and Surfactant protein C (*Sftpc*), Glyceraldehyde 3-phosphate dehydrogenase (*Gapdh*) was utilized as a housekeeping gene, RNA was reverse transcribed using iScript cDNA synthesis kit (Bio-Rad, Hercules, CA) and RT-qPCR analysis was performed using iTaq SYBR Green supermix (Bio-Rad) in an Applied Biosystems 7,500 multicolor real-time PCR detection system (Applied Biosystems), as before (Bernau et al., 2021). The following SYBR Green primer sequences were used: *Tnc* forward: ACC ATGCTGAGATAGATGTTCCAAA, *Tnc* reverse: CTTGAC AGCAGAAACACCAATCC; *Sftpc* forward: GTAGCAAAG AGGTCCTGATG, *Sftpc* reverse: CCTACAATCACCACGACAA; *Gapdh* forward: GCCCAAGATGCCCTTCAGTG, *Gapdh* reverse: CATCCACTGGTGCTGCCAAG. Prior to experimental use, standard curves and primer efficiencies were determined for the primer pairs above. Following all experimental RT-qPCR, relative changes in gene expression were quantified compared to control housekeeping gene using the ΔΔCt method.

### Bronchoalveolar Lavage

Bronchoalveolar lavage (BAL) fluid was extracted from mouse lungs as before (Bernau et al., 2015; Bernau et al., 2021). Following anesthetization and exsanguination, mice were intubated by inserting a catheter into the exposed trachea. Lungs were lavaged five times with 0.8–1 ml of Dulbecco’s phosphate-buffered saline (DPBS, Corning Inc., Corning, NY), and BAL cells isolated, counted and cytospins prepared. Wright Giemsa staining (Fisher Scientific, Hampton, NH) was performed on the cytospins and differentials were counted by blinded observers to determine the ratio of neutrophils out of a total of 200 counted cells.

### Statistical Analyses

Logrank test was utilized for statistical analysis of mouse mortality. Two-way Analysis of Variance (ANOVA) with Sidak post-hoc test was utilized for analyses of mouse groups compared to voxel HU range or weight-loss. Pearson’s product-moment correlation analysis was utilized to establish a relationship between αSMA IHC quantification and hydroxyproline content, Ashcroft scores, μCT densitometry (voxels between −200 and 100 HU) or blood O_2_ saturation, as well as the relationship between blood O_2_ saturation and μCT densitometry (voxels between −200 and 100 HU and −500 to −400 HU). All other data were analyzed using Student’s *t*-test (to compare two conditions) or One-Way ANOVA with Holm-Šídák post-hoc test (to compare more than two conditions). For all analyses, *p* < 0.05 was considered statistically significant.

## RESULTS

In order to test our hypothesis, we employed a murine model of bleomycin-induced lung injury. This is a well-established model of pulmonary fibrosis that produces several distinct phases: acute inflammation (day 1–7), fibrosis (day 8–28), followed by resolution (day 28 and later) (Moeller et al., 2008). To exclude artifacts of BRD4 inhibition on the inflammatory response, we employed a rigorous therapeutic design strategy (Moeller et al., 2008; Jenkins et al., 2017), commencing the administration of ZL0591 (10 mg/kg/day) during the established “fibrotic” phase (14 days post-bleomycin) (Izbicki et al., 2002).

### Treatment With ZL0591 After Established Pulmonary Fibrosis Improves Blood O_2_ Saturation

**FIGURE 1 |:**
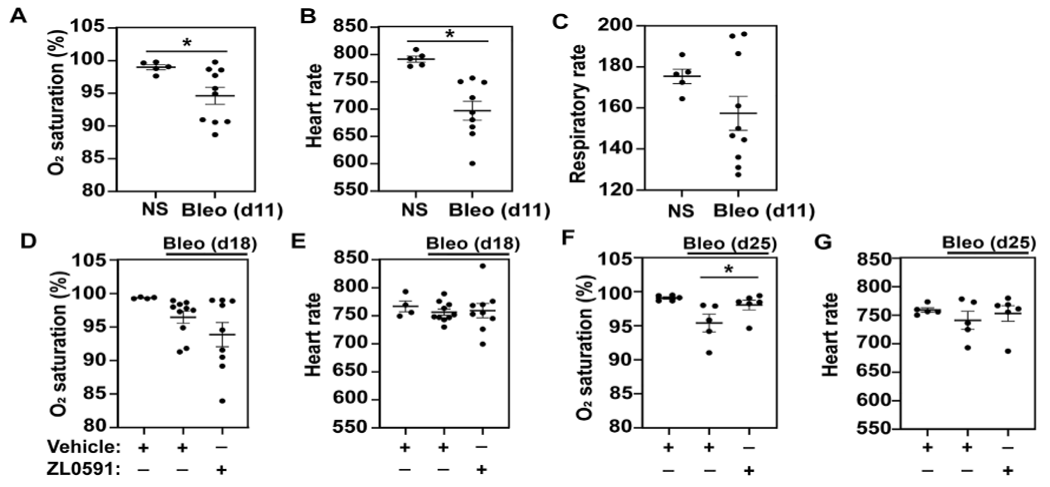
BRD4 BD1 inhibitor improves blood O_2_ saturation upon bleomycin injury. On day 0, mice were intratracheally treated with bleomycin (bleo, 1 U/kg) or normal saline (NS) control. ZL0591 (10 mg/kg, IP), or vehicle control, was administered intraperitoneally starting on day 14 daily until day 21 and every other day until day 28. **(A–C)** On day 11, mouse vital signs were collected, including blood O_2_ saturation, heart rate, and respiratory rate (as indicated). Unpaired Student’s *t*-test was used to assess statistical significance (*p < 0.05). **(D–G)**. Blood O_2_ saturation and heart rate were measured again 18 and 25 days after bleomycin (as indicated). One-way ANOVA with šidák correction for multiple comparisons was used for statistical testing (*p < 0.05). Data represent *n* = 4–10 mice/condition and are depicted by scatter plots with mean ± SEM.

In order to monitor the effects of ZL0591 longitudinally in live mice, each week we collected serial blood O_2_ saturations via pulse oximetry, as well as heart and respiratory rates. We first obtained these measurements on day 11 post-bleomycin injury, 3 days prior to commencing treatment with ZL0591, in order to determine the baseline differences between bleomycin-treated animals and controls. At this point, we found that blood O_2_ saturation and heart rate were reduced in the bleomycin-treated group compared to uninjured controls, while the respiratory rate was unchanged between the two groups (Figures 1A–C). After initiation of ZL0591 administration, we continued to collect vital sign measurements weekly (on days 18 and 25). On day 18 (4 days after initiation of ZL0591 treatment), we did not observe any differences in the bleomycin/ZL0591 group compared to the bleomycin/vehicle controls (Figures 1D,E). In contrast, by day 25 (11 days after initiation of ZL0591 treatment and during the phase of mature fibrosis), we found a significant improvement in blood O_2_ saturation in the bleomycin group treated with ZL0591 compared to bleomycin/vehicle controls (Figure 1F), consistent with an improvement in gas exchange. We did not observe a change in the heart rate as a result of ZL0591 treatment at this time point (Figure 1G). The observed improvement in blood O_2_ saturation provided a real-time indication that treatment with ZL0591 may be modifying components of the fibrotic response.

### Treatment With ZL0591 Attenuates Established Bleomycin-Induced Pulmonary Fibrosis

**FIGURE 2 |:**
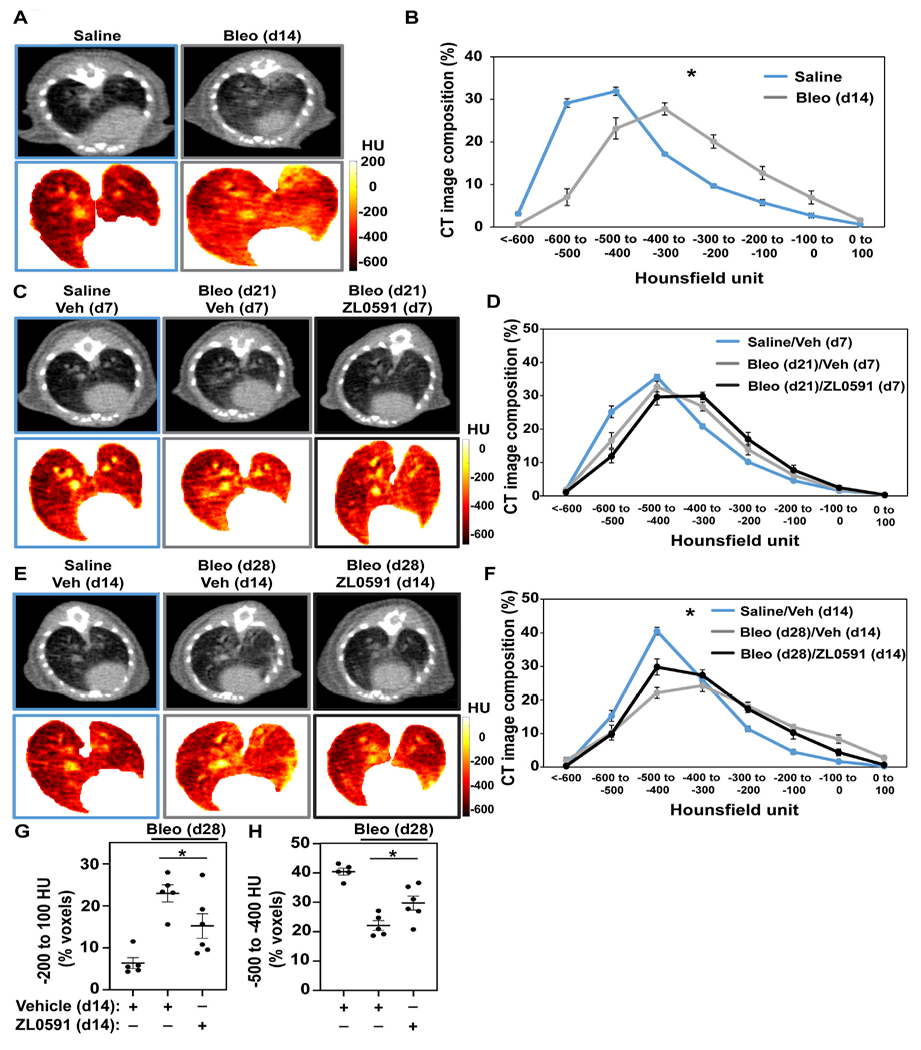
Micro CT analysis indicates reduced fibrotic response as a result of ZL0591 treatment in bleomycin injury. Mice were treated intratracheally with bleomycin (bleo, 1 U/kg) or normal saline (NS) control on day 0. ZL0591 (10 mg/kg, IP), or vehicle control, was administered intraperitoneally starting on day 14 daily until day 21 and every other day until day 28 post-bleomycin. Fourteen **(A,B)**, 21 **(C,D)**, and 28 **(E,F)** days after bleomycin instillation, mice were imaged *via* μCT (105 μm resolution). Representative μCT images and corresponding Hounsfield unit (HU) heat maps are displayed for each time point **(A,C,E)**. Micro CT lung tissue density was analyzed by measuring HU in each lung voxel (excluding surrounding tissue) and subsequently determining the spread of the voxels across different categories of HU thresholds **(B,D,F)**. Grouped data were analyzed at each time point using Two-way ANOVA (*p < 0.05) with šidák correction for multiple comparisons. Data represent *n* = 4–10 mice/condition and are depicted by line charts (mean ± SEM). **(G,H)** Highest radiodensity range voxels (−200 to +100) were combined to assess extent of fibrosis **(G)**, while −500 to −400 HU voxel range was analyzed to evaluate improvement in lung aeration **(H)**. One-way ANOVA was utilized for statistical analysis in **(G,H)** (*p < 0.05). Data represent *n* = 5–6 mice/condition and are depicted by a scatter plot with mean ± SEM.

The same treatment groups then underwent μCT imaging of the lungs to provide longitudinal observations of the fibrotic response (Scotton et al., 2013). At day 14 (prior to treatment with ZL0591), we observed an increase in radiodensity indicative of parenchymal fibrosis in bleomycin-treated lungs (Figure 2A), consistent with previous observations (Scotton et al., 2013; Ruscitti et al., 2017). Analysis of the distribution of voxel-based HU from volumetric analysis of lung μCT images is shown in Figure 2B. These data show a shift in the distribution of HU in the lung images from bleomycin-treated animals compared to controls, with a decrease in low-attenuation HU voxels (which correspond to air), and an increase in high-attenuation voxels corresponding to fluid/tissue. These results are consistent with an increase in soft-tissue density within the lung parenchyma from either edema or fibrosis, for example. μCT imaging of control- and ZL0591-treated groups was then performed on day 21 post-bleomycin (7 days into ZL0591) treatment. At this time point, we did not observe an overall change in radiodensity between the two bleomycin-treated groups (Figures 2C,D). However, μCT imaging at day 28 post-bleomycin (and 14 days into the ZL0591 treatment) showed a leftward shift in voxel HU distribution in the bleomycin/ZL0591-treated group compared to bleomycin-treated control animals, indicating a significant improvement in radiodensity, and thus diminished fibrosis, in this group (Figures 2E,F). Corroborating this finding, analysis of the highest attenuation HU, voxels from the μCT images (−200 to +100) that correspond with soft-tissue density (i.e., fibrosis), showed that bleomycin/ZL0591-treated mice had significantly fewer voxels in this range compared to bleomycin/control-treated animals (Figure 2G). It should be noted that we also observed that the percentage of high HU voxels inversely correlated with blood O_2_ saturation (Supplementary Figure S1A). Furthermore, we found an increased voxel percentage in the–500 to −400 HU range under treatment with ZL0591 (Figure 2H), indicative of improvement in lung aeration. Notably, the percentage of voxels in this range positively correlated with blood O_2_ saturation (Supplementary Figure S1B). Taken together, the μCT imaging data demonstrate that 14 days after treatment with ZL0591 (day 28 post-bleomycin injury), there was a decrease in μCT lung opacification (higher voxel HU), consistent with attenuation of overall fibrosis.

**FIGURE 3 |:**
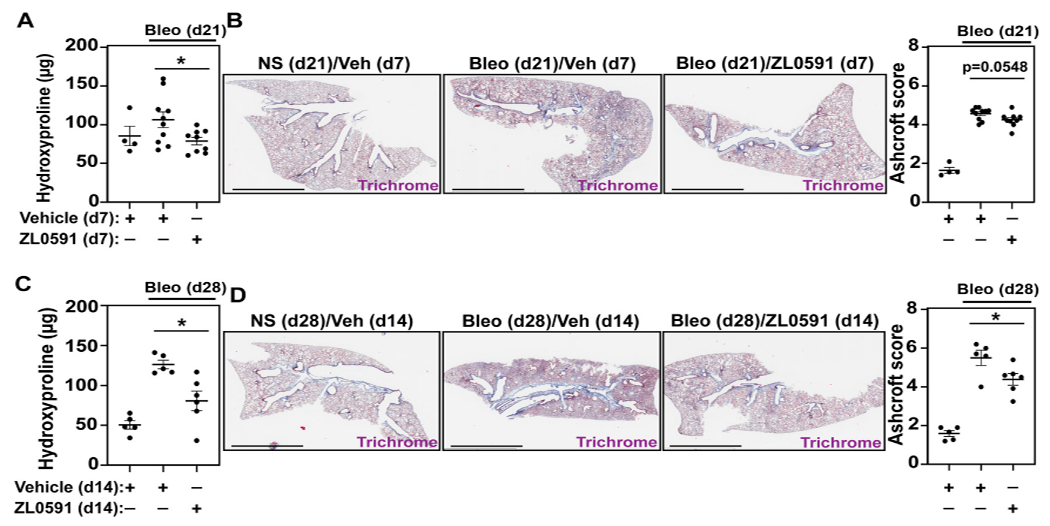
ZL0591 treatment decreases collagen accumulation in murine model of pulmonary fibrosis. Mice were treated intratracheally with bleomycin (bleo,1 U/kg) or normal saline (NS) control on day 0. ZL0591 (10 mg/kg, IP), or vehicle control, was administered daily from day 14 until 21 and every other day thereafter until day 28 post-bleomycin. **(A,C)** Collagen accumulation in the superior, inferior and post-caval lobes of the right lungs was assessed via hydroxyproline assay, 21 and 28 days post-bleomycin instillation. **(B,D)** Left lungs were inflated and fixed in 4% neutral buffered formalin, paraffin embedded and stained with Masson Trichrome staining. Representative images are displayed and collagen content was quantified using modified Ashcroft scoring in approximately 10 fields of view at ×20 magnification from lungs collected 21 and 28 days post-bleomycin. Scale bar = 3 mm. One-way ANOVA was used for assessment of statistical significance with šidák correction for multiple comparisons (*p < 0.05). Data represent *n* = 4–10 mice/condition and are depicted by scatter plots with mean ± SEM.

We then analyzed mouse lungs from each of the experimental groups described above to assess alterations in bleomycin-induced collagen deposition after treatment with ZL0591. Mouse right lung lysates were subjected to hydroxyproline assay to determine collagen content. In mice treated with ZL0591 starting 14 days after bleomycin injury and euthanized at day 21 (7 days of ZL0591 treatment), we observed a significant decrease in lung collagen content in comparison to mice treated with the vehicle control at the same time point (Figure 3A). Left lungs from the same mice were fixed, sectioned, and subjected to Masson’s trichrome staining, followed by semi-quantitative histologic scoring for fibrosis, using a modified Ashcroft method (Hübner et al., 2008). At this time point, we did not observe a significant difference in Ashcroft scores, but a trend was present (*p* = 0.0548) (Figure 3B). Given the observed trend toward reduced fibrosis after a 7-day treatment with ZL0591, we hypothesized that increased treatment duration may result in attenuation of the fibrotic response.

Thus, on day 28 after bleomycin injury and 14 days after initiation of ZL0591 treatment, we again determined hydroxyproline levels from lung lysates. In this case, we observed a similar, significant decrease in hydroxyproline in lungs from bleomycin-injured mice treated with ZL0591 compared to vehicle control, consistent with a reduction in collagen content (Figure 3C). In addition, Ashcroft scoring of histologic sections from left lungs indicated a reduction in fibrosis (Figure 3D), consistent with the decrease in collagen content that we observed at this time point. Taken together, these results suggest a time-dependent improvement in the amount of total fibrosis in response to ZL0591 treatment.

### ZL0591 Treatment Decreases Myofibroblast Expansion in Bleomycin-Induced Pulmonary Fibrosis

**FIGURE 4 |:**
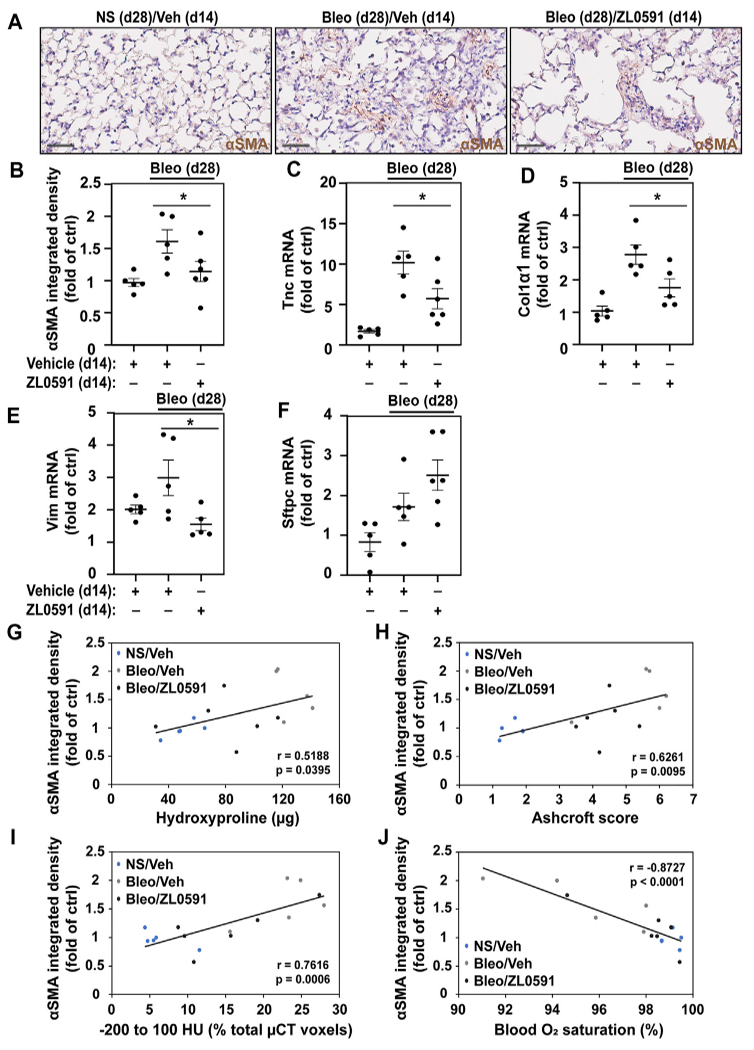
ZL0591 inhibits myofibroblast expansion in bleomycin model of pulmonary fibrosis. Mice were intratracheally treated with bleomycin (bleo, 1 U/kg) or normal saline (NS) on day 0, followed by intraperitoneal ZL0591 (10 mg/kg, IP),or vehicle control, treatment daily from day 14 until 21 and every other day until day 28. **(A)** Left lungs were inflated and fixed in 4% neutral buffered formalin, paraffin embedded and immunohistochemically stained against αSMA (brown). Representative images are displayed. Scale bar = 50 μm. **(B)** Brown (αSMA) staining was quantified in approximately 11 fields of view at ×40 magnification avoiding large airways and vessels. **(C–F)** Middle lobes of the right lungs were lysed for total mRNA and subjected to RT-qPCR against tenascin (*Tnc*) **(C)**, collagen 1α1 (*Col1α1*) **(D)**, vimentin (*Vim*) **(E)**, surfactant protein C (*Sftpc*) **(F)** and a housekeeping gene. **(B–F)** One-way ANOVA was used for assessment of statistical significance with šidák correction for multiple comparisons (*p < 0.05). Data represent *n* = 5–6 mice/condition and are depicted by a scatter plot with mean ± SEM. αSMA IHC quantification of samples collected 28 days post-bleomycin was correlated against total hydroxyproline **(G)**, Ashcroft scores **(H)**, μCT densitometry [voxels between −200 and 100 HU; **(I)**] and blood O_2_ saturation **(J)** atthe same time points. Pearson’s correlation was utilized for statistical analyses in **(G–J)** (*p < 0.05). Data represent *n* = 5–6 mice/condition and are depicted by a scatter plot with a linear trendline.

To ascertain whether the decreased fibrotic response was related to ZL0591s effect on the formation of a myofibroblast population, we utilized αSMA IHC staining of sectioned mouse lungs. We observed a loss of the myofibroblast marker, αSMA, in bleomycin/ZL0591-treated mouse lungs compared to bleomycin/vehicle controls at day 28 post-bleomycin (14 days following the start of ZL0591 treatment) (Figures 4A,B). This was consistent with a reduction in the myofibroblast population. At the same time point, we also found a reduction in bulk transcript levels of the mesenchymal marker *Vim*, along with reduction in myofibroblast associated ECM genes *Tnc* and *Col1α1* (Figures 4C–E). These data support global downregulation of myofibroblast-associated genes by ZL0591. On the other hand, we did not detect a significant change in the transcription of markers of other cell types, including alveolar epithelial type 2 cell (AEC2) marker, *Sftpc*, suggesting that this cell population may be preserved (Figure 4F). Importantly, there was a significant correlation between myofibroblast presence (αSMA-positive IHC signal) and increased fibrotic burden, based on regression analysis of hydroxyproline, Ashcroft scoring, μCT densitometry (voxels between −200 and 100 HU) and (low) blood O_2_ (Figures 4G–J). Altogether, these data indicate that inhibition of BRD4 activity by ZL0591 diminishes bleomycin-induced pulmonary fibrosis, possibly due to inhibition of myofibroblast transdifferentiation.

### ZL0591 Does Not Impact the Inflammatory Response in the Fibrotic Phase of Bleomycin Injury

**FIGURE 5 |:**
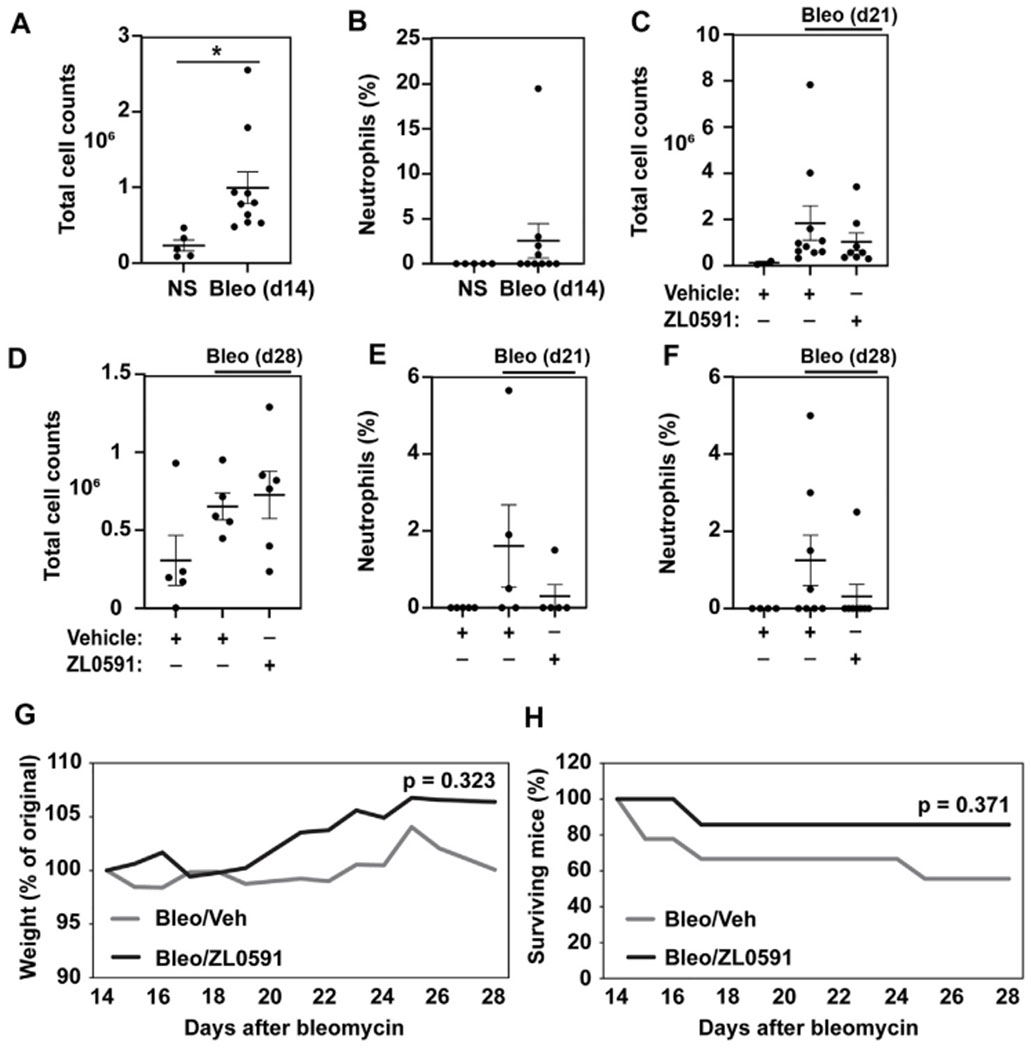
Delayed administration of ZL0591 does not impact the inflammatory response in bleomycin injury. Mice were treated with intratracheal bleomycin (bleo, 1 U/kg) or normal saline (NS) control on day 0, followed by intraperitoneal injections of ZL0591 (10 mg/kg) daily from day 14 until 21 and every other day thereafter until day 28. **(A,C,D)** Total bronchoalveolar lavage cells were counted at indicated time points. **(B,E,F)** Percent of neutrophils was calculated from total bronchoalveolar lavage cell counts at indicated time points. **(A,B)** Unpaired Student’s *t*-test was used to assess statistical significance (*p < 0.05) in data collected at day 14 post-bleomycin injury. **(C–F)** One-way ANOVA utilizing šidák correction for multiple comparisons (*p < 0.05) was used for statistical analyses of data collected at day 21 and 28 post-bleomycin injury. Data represent *n* = 4–10 mice/condition and are depicted by scatter plots with mean ± SEM. **(G)** Mouse weights were collected daily and plotted as percent of original. Two-way ANOVA was utilized for assessment of statistical significance between the two bleomycin-treated groups over time. **(H)** Percent survival in each group over the course of the experiment. Log rank test was used to assess statistical significance. Data represent *n* = 5–9 mice/condition.

BRD4 is a multifunctional enzyme that is also implicated in the inflammatory response. Thus, we sought to assess whether our treatment approach had an effect on this aspect of the bleomycin injury. The inflammatory response to a single-dose intratracheal bleomycin-induced lung injury is most pronounced 3 to 7 days later, with increased numbers of total BAL cells and neutrophilia. In our study, we observed elevated numbers of BAL cells 14 days after bleomycin administration (Figure 5A), but an absence of a neutrophilic response (Figure 5B). By days 21 and 28 post-bleomycin injury, the total number of BAL inflammatory cells in bleomycin and vehicle treated mice was no longer increased compared to uninjured NS vehicle controls, and ZL0591 treatment did not modify this response (Figures 5C,D). At these time points, neutrophilic inflammation was low and we did not detect any significant ZL0591-induced changes at this late stage of bleomycin-induced fibrosis (Figures 5E,F). Finally, we assessed weight loss and survival after bleomycin administration. We found that initiation of ZL0591 treatment 14 days after bleomycin injury did not impact animal weight loss from that time point (Figure 5G). Similarly, we did not observe an effect of ZL0591 treatment on mouse mortality after initiation of treatment (Figure 5H).

## DISCUSSION

BRD4 is a member of the BET family that serves as a multifunctional chromatin regulator and plays key roles in cell cycle progression, DNA damage-repair, innate inflammation and cell-state transition processes driving oncogenic transformation and hypertrophic diseases (Devaiah et al., 2016b; Donati et al., 2018). Of specific relevance to this study, BRD4 mediates transcriptional elongation, controlling genes important in TGFβ-induced myofibroblast transition. In this study, we used our novel, highly BD-selective chemistries to assess whether BRD4 BD1 plays a role in maintenance of bleomycin-induced fibrosis in mice. Specifically, we found that inhibition of BRD4 BD1 after establishment of the fibrotic response (14 days post-bleomycin), results in attenuation and reversal of total lung fibrosis by day 28. This was evidenced by reduction in collagen content and myofibroblast presence, as well as improvement in gas exchange.

The bleomycin model of pulmonary fibrosis is the most frequently used and widely accepted model for preclinical studies of IPF (Jenkins et al., 2017), and has been extensively utilized for preclinical studies investigating medications currently employed to treat this disease (Chaudhary et al., 2007; Inomata et al., 2014). One currently approved medication, pirfenidone, is a small molecule compound that exhibits anti-fibrotic and anti-inflammatory properties and was approved in 2008 for treatment of mild-moderate IPF (Spagnolo et al., 2010). While some preclinical rodent studies analyzed the effects of prophylactic pirfenidone administration (Oku et al., 2008; Inomata et al., 2014), the “proof of efficacy” experimental design, coinciding pirfenidone treatment with bleomycin administration, complicates separation of anti-inflammatory activity from anti-fibrotic treatment (Jenkins et al., 2017). One mouse study assessed pirfenidone’s efficacy starting 14 days after the commencement of five daily intravenous bleomycin injections and found a reduction in collagen content and αSMA-expressing myofibroblast population in the pirfenidone-treated group (Kakugawa et al., 2004). However, more recent systematic reviews have concluded that, although the currently available FDA-approved IPF medications reduce the loss of vital capacity, there is a high side-effect burden and it is less clear that mortality is reduced (Skandamis et al., 2019). The need for more effective treatments has stimulated the search and evaluation of new classes of IPF therapeutics, such as those targeting BET proteins. In one seminal study, it was reported that oral administration of JQ1 (at 200 mg/kg/d) given 5 days after intratracheal bleomycin administration attenuated myofibroblast accumulation, reduced hydroxyproline accumulation by 50%, and reduced histological evidence of fibrosis (Tang et al., 2013). However, this study was limited in a number of ways. First, JQ1 reduced macrophage and neutrophil numbers, a finding consistent with the inhibitor being administered during the active inflammatory phase, and was thus, not truly a “therapeutic” experimental design. Second, the dose of JQ1 is well-known to cause toxicity, resulting in bone marrow suppression (Lee et al., 2016), making the understanding of its effects on pulmonary inflammation ambiguous. Third, the nonselectivity of JQ1 precludes an understanding of which BET protein mediates fibrosis, along with the potential of inducing off-target effects. Our study advances the field through a rigorous therapeutic experimental design using a highly selective BRD4 inhibitor that engages the BRD4 BD1 in a novel site (Liu et al., 2022), and lacks evidence of hematologic supression (Liu et al., 2018).

The focus on BRD4 as a therapeutic target is supported by mechanistic studies that demonstrate its role in myofibroblast transdifferentiation. BRD4 regulates myofibroblast transition by controlling transcriptional elongation, a multistep process mediated by activation of genes within an open chromatin environment (Yang et al., 2017). Recent applications of protein profiling of the BRD4 complex have elucidated that BRD4 is a core of a dynamic multiprotein complex containing hundreds of transcription factors, cyclin dependent kinases, and chromatin modifying proteins (Zhang et al., 2017; Mann et al., 2021). BRD4 forms these multi-subunit complexes through several distinct domains. For example, BRD4 interacts with acetylated histones and transcription factors through its BDs, with transcriptional regulators through its extraterminal domain, and with transcriptional elongation kinases through its carboxyterminal domain (Bisgrove et al., 2007; Rahman et al., 2011; Mann et al., 2021). The recent development of highly selective small-molecule BRD4 BD inhibitors have enabled us to probe the role of BRD4 BD interactions in complex biological models associated with epithelial inflammation, trained immunity and cell-state changes, suggesting that BRD4 plays a central role in host defense to RNA viruses (Tian et al., 2016; Chang et al., 2016; Tian et al., 2019b; Wang et al., 2020). Here, we explore the role of the BRD4 BD1 in myofibroblast transdifferentiation in a mouse model of interstitial pulmonary fibrosis.

Previous work demonstrated that despite cooperation between the BET proteins, BRD4 appears to hold primary responsibility for steady-state gene expression maintenance (Gilan et al., 2020) and is required for the transition of quiescent fibroblasts towards a pro-fibrotic mesenchymal phenotype (Tian et al., 2016). BRD4 regulates expression of Nox4, an enzyme required for TGFβ-induced production of reactive oxygen species and fibroblast to myofibroblast transition in pulmonary fibrosis (Hecker et al., 2009; Amara et al., 2010; Hecker et al., 2014). JQ1, a nonselective competitive inhibitor of the BET binding pocket, has previously been shown to attenuate TGFβ-induced Nox4 overexpression, oxidative stress and myofibroblast activity *in vitro*, as well as bleomycin-induced pulmonary fibrosis in mice (Tang et al., 2013; Stock et al., 2019). However, since JQ1 indiscriminately blocks the high affinity binding pockets of BRD2, BRD3, as well as both BD1 and BD2 of BRD4, it could not be utilized for assessment of the role of different BET family members or BDs in the fibrotic process. For this reason, we previously developed an array of selective inhibitors and utilized ZL0591, a selective allosteric inhibitor that targets BRD4 BD1 with ~100 nM affinity, while demonstrating low affinity for BRD4 BD2, BRD2 and BRD3, to enable elucidation of the role of BRD4 BD1 in pulmonary fibrosis (Liu et al., 2020; Liu et al., 2022). We found that administration of ZL0591 during the fibrogenic stage of bleomycin-induced injury in mice attenuates fibrosis, demonstrating that BRD4 acts through its BD1 and is the member of the BET family responsible for mediating these pro-fibrotic effects. As such, this report adds to our understanding of the contribution of bromodomain-containing proteins in tissue remodeling and fibrosis.

In this study we utilized a single-dose intratracheal bleomycin instillation as a model for pulmonary fibrosis, characterized by early alveolar epithelial cell injury and inflammation that gives way to the pro-fibrotic phenotype starting around 8 days post-bleomycin (Izbicki et al., 2002; Moeller et al., 2008). While the bleomycin model of murine pulmonary fibrosis is commonly utilized for preclinical studies of this disease, the initial inflammatory phase that precedes onset of fibrosis is one of its major limitations. In order to focus on the fibrotic phase of the model, our study addressed this limitation by administering ZL0591 starting 14 days after bleomycin instillation, during the peak of the fibrotic response and past the expected stage of intense epithelial cell injury and inflammation. Importantly, we demonstrate here that BRD4 BD1 plays a critical role during the fibrotic stage of bleomycin injury, while the timeline of ZL0591 administration strengthens our findings, confirming that its effects are not secondary to attenuation of the initial inflammatory phase of bleomycin injury.

Previous work using non-BET selective BD1 and BD2 inhibitors showed the BET BD1 domain to be important in oncogenic potential, whereas BD2 was important in inflammation (Gilan et al., 2020). Here, we extend this observation to the BRD4 BD1 and find that administration of ZL0591 during the fibrotic phase of the bleomycin-induced injury had no effect on inflammation, which was notable given the critical role of BRD4 in the innate inflammatory response (Brasier et al., 2011; Tian et al., 2019b). Specifically, our previous work demonstrated a vital role for BRD4 in TGFβ/NFκB-mediated mesenchymal transition, remodeling and fibrosis upon repetitive TGFβ administration in mice (Tian et al., 2016). Our present study aimed to elucidate the role of BRD4 BD1 during the fibrotic stage of bleomycin injury by administering ZL0591 14–28 days post-bleomycin treatment, which is not characterized with high levels of cellular inflammation. Confirming this, we found no significant neutrophilia or increased BAL cell counts by day 21 and 28 post-bleomycin instillation compared to NS-treated controls. As such, BAL cell quantification could not be used to determine the effects of ZL0591 on broader bleomycin-induced inflammatory signaling at these fibrotic time points. The consequences of ZL0591 administration that we report here may, nevertheless, be mediated through an alteration in inflammatory signaling, either directly through decreased TGFβ/NFκB-induced mesenchymal transition or indirectly by disrupting the release of epithelial mediators required for myofibroblast differentiation, such as TGFβ. Examining these effects will be the focus of future investigations.

The mechanisms behind how selective BRD4 BD inhibitors disrupt epigenetic control are just beginning to be understood. We recently found that inhibition of BRD4 BDs disrupt association of more than 200 transcription factors in the activated complex (Mann et al., 2021), and that BRD4 inhibitors block expression of key components of transcriptional coactivators, chromatin remodeling proteins as well as BRD4 itself (Xu et al., 2021). Consequently, follow-up studies are needed to determine the mechanisms by which ZL0591 disrupts and reverses bleomycin-induced fibrosis in mice.

Recent single cell RNA sequencing studies identified *Tnc* as a highly specific transcript expressed by myofibroblasts (Tsukui et al., 2020). Our present study reports a reduction of *Tnc* transcription, in addition to decreased αSMA expression in the lung parenchyma, in the ZL0591-treated mice, strongly supporting the conclusion that ZL0591 treatment reduces the population of pathogenic myofibroblasts. While we demonstrate a correlative relationship between myofibroblast loss and attenuation of fibrosis, determining the sequence of these events is beyond the focus of the present study. For example, in this model, TGFβ-induced Nox4 signaling could be dependent on Smad3 recruitment of BRD4, as is the case in dermal fibroblasts during wound healing (Ijaz et al., 2017). Furthermore, while we did not observe a broad change in the inflammatory response or an effect on non-fibroblast cell types upon ZL0591 treatment in this initial study, additional adequately powered studies are needed to interrogate these effects further and mechanistically assess the impact of ZL0591 administration in bleomycin-induced fibrosis.

In conclusion, here we show that BRD4, an epigenetic regulator that serves as a scaffold for nuclear activators and transcription factors, is critical in the progression of bleomycin-induced fibrosis and myofibroblast expansion in mice. These findings are corroborated by evidence of improved gas exchange in mice treated with the BRD4 BD1 inhibitor, ZL0591. This study provides the essential pre-clinical evidence for reversal of fibrosis using this epigenetic target that can lead to important clinical advancements for IPF.

## Supplementary Material

Supplementary Material

## Data Availability

The raw data supporting the conclusion of this article will be made available by the authors, without undue reservation.
