# Supplementary material for: Selective Inhibition of Bromodomain-Containing Protein 4 Reduces Myofibroblast Transdifferentiation and Pulmonary Fibrosis
Source: Front Mol Med. Author manuscript; Available in PMC 2022 Jun 30. (PMC9245900; doi:10.3389/fmmed.2022.842558)
Supplement: Supplementary file 1 [file NIHMS1810153-supplement-Supplementary_Material.pdf]

Supplementary Material

1.1 Supplementary Figures

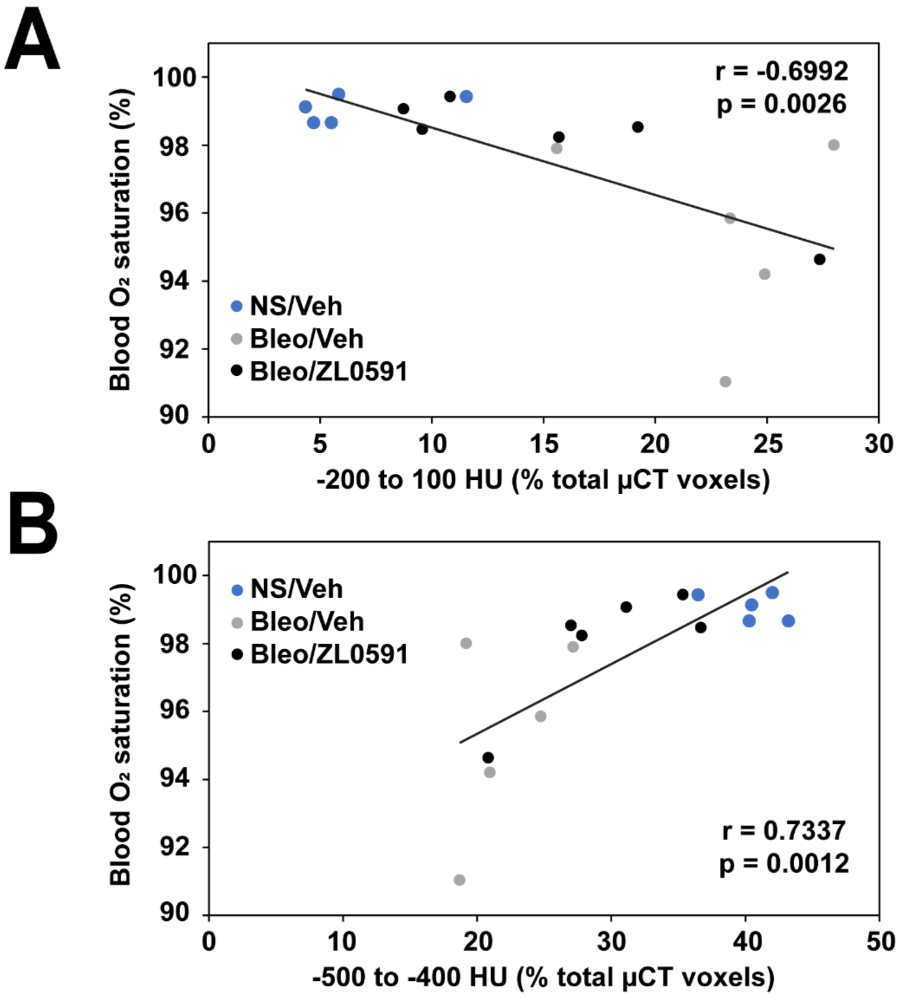

Supplementary Figure 1. Improvement in blood O_2 saturation inversely correlates with micro CT-detected lung fibrosis in mice. Mice were intratracheally treated with bleomycin (bleo, 1 U/kg) or normal saline (NS) on day 0, followed by intraperitoneal ZL0591 (10 mg/kg, IP), or vehicle control, treatment daily from day 14 until 21 and every other day until day 28. On day 25 post-bleomycin treatment, mouse blood O_2 saturation was measured, followed by micro CT imaging three days later. Micro CT lung tissue density was analyzed by measuring HU in each lung voxel (excluding surrounding tissue) and determining the spread of the voxels across different categories of HU thresholds. Blood O_2 saturation was correlated with micro CT voxels associated with severe fibrosis (-200 to +100 HU) **(A)**, and voxels associated with improved lung aeration (-500 to -400 HU) **(B)**. Pearson's correlation was utilized for statistical analyses. Data represents n = 5-6 mice/condition and is depicted by a scatter plot with a linear trendline.
